# Silencing of long noncoding INHBA antisense RNA1 suppresses proliferation, migration, and extracellular matrix deposition in human hypertrophic scar fibroblasts via regulating microRNA-141-3p/myeloid cell leukemia 1 axis

**DOI:** 10.1080/21655979.2021.1919013

**Published:** 2021-05-12

**Authors:** Yan Yang, Chun Xiao, Kang Liu, Liping Song, Yonggang Zhang, Birong Dong

**Affiliations:** a Sichuan Chidingshengtong Biotechnology Co., Ltd., Chengdu, Sichuan, P.R. China; b E’mei Mountainside Health Management Co.Ltd., Chengdu, Sichuan, P.R. China; c National Clinical Research Center of Geriatrics, West China Hospital, Sichuan Universtiy, Chengdu, Sichuan, P.R. China; d Institute of Tissue Engineering and Stem Cells, Nanchong Central Hospital, the Second Clinical Institute of North Sichuan Medical College, Nanchong, Sichuan, P.R. China; e Nanchong Key Laboratory of Cancer Biotherapy, Nanchong, Sichuan, P.R. China; f Center for Stem Cell Research and Application, Institute of Blood Transfusion, Chinese Academy of Medical Sciences&Peking Union Medical College (CAMS&PUMC), Chengdu, Sichuan, P.R. China

**Keywords:** Hypertrophic scar, INHBA-AS1, proliferation, migration, extracellular matrix deposition

## Abstract

Long noncoding RNAs (lncRNAs) play vital roles in the progression of hypertrophic scar (HS). We aimed to explore the effect of lncRNA INHBA Antisense RNA1 (INHBA-AS1) in the formation of HS and identify the potential mechanisms. INHBA-AS1 and microRNA (miR)-141-3p expression in human HS fibroblasts (hHSFs) was determined using RT-qPCR. LncBase online database predicted that miR-141-3p could be a putative target of INHBA-AS1, and the interaction of them was verified by luciferase reporter assay and RNA immunoprecipitation (RIP) assay. Subsequently, following INHBA-AS1 silencing, cell proliferation and migration were evaluated using CCK-8, wound healing and Transwell assays. And rescue experiments were conducted to analyze the impact of INHBA-AS1 and miR-141-3p on HS formation. Immunofluorescence assay was employed to examine the expression of extracellular matrix (ECM)-related proteins. Then, StarBase database predicated that myeloid cell leukemia 1 (MCL1) was a potential target of miR-141-3p, which was verified with luciferase reporter- and RIP assays. Finally, cell function and ECM deposition were determined after MCL1-downregulation. INHBA-AS1 was significantly elevated while miR-141-3p was notably reduced in hHSFs. And it was confirmed that miR-141-3p was directly targeted by INHBA-AS1. Moreover, INHBA-AS1 silencing markedly attenuated the proliferation, migration and ECM accumulation of hHSFs, which were restored after miR-141-3p silencing. Additionally, MCL1 was confirmed as a direct target of miR-141-3p, and MCL1-knockdown remarkably alleviated the proliferation, migration and ECM accumulation of hHSFs. INHBA-AS1-knockdown suppresses the formation of HS by regulating miR-141-3p/MCL1 pathway, suggesting a promising therapeutic target for HS treatment.

## Graphical Abstract

**Figure:**
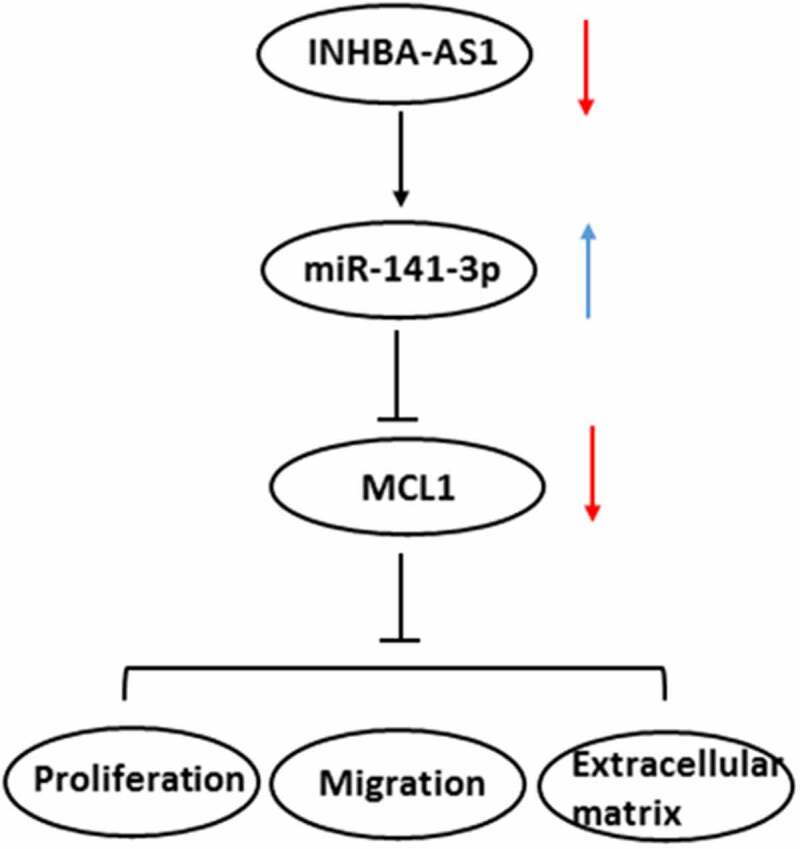

## Introduction

Hypertrophic scar (HS), a pathogenic form of scar formation, is frequently observed after the healing of wounds on skin caused by burns, lacerations and surgeries [1,2]. A definitive treatment for HS has not formed yet so far, although many treatment options available for HS, such as surgery, steroid injections, laser and skin grafting [3]. Thus, identifying new effective therapeutic targets and investigating the detailed mechanisms in the occurrence and development of HS are essential for treatment of this disease.

Substantial evidence exists to suggest that the pathogenesis of HS is closely involved in abnormally increased activity and over-proliferation and migration of fibroblasts, as well as the superabundant accumulation of extracellular matrix (ECM) component [4,5]. Collagen I (Col I), collagen III (Col III), collagen IV (Col IV) and alpha smooth muscle actin (α-SMA) levels are visibly intensified during HS, which together play significant roles in the fibrotic ECM environments [6,7]. Accumulating study confirms that suppression of HSF proliferation, migration and ECM accumulation relieve HS development [8,9]. Long non-coding RNA (lncRNA) is a novel type of noncoding RNAs with limited or no protein-coding ability and over 200 nucleotides in length [10]. Numerous studies have unveiled that lncRNAs exert significant effects on pathological processes [11]. A considerable body of evidence indicates that lncRNAs can regulate multiple physiopathological processes by binding to microRNAs (miRNAs) [12,13]. Compelling evidence revealed that lncRNAs are related to the development of HS [10,14]. Among these lncRNAs, INHBA-AS1 was reported to be elevated significantly in several human tissues sample. For instance, INHBA-AS1 is enriched in human gastric cancer tissues and significantly elevated in the plasma of patients with gastric cancer [15]. INHBA-AS1 is highly expressed in colorectal cancer tissues and cells, and NHBA-AS1 silencing suppresses the proliferation of colorectal cancer cells [16]. INHBA-AS1-upregulation promotes cell growth, migration and invasion progression of oral squamous cell carcinoma [17]. Importantly, compelling evidence indicates that INHBA-AS1 is overexpressed in human hypertrophic scar tissues compared with the normal skin tissues by high-throughput RNA sequencing [18]. However, the roles of INHBA-AS1 in the development of HS remain unknown.

The aim of the present study is to explore the effects of INHBA-AS1 on the proliferation, migration and ECM accumulation of human HS fibroblasts (hHSFs), and to clarify its regulatory effects on miR-141-3p and myeloid cell leukemia 1 (MCL1).

## Materials and methods

### Cell culture

Human embryonic skin fibroblasts (CCC-ESF-1) was provided by Shanghai Zibo Biological Technology Co., Ltd (cat no. YB-aTcc-3084; Shanghai, China). hHSFs were the product of Shanghai Guandao Biological Engineering Co., Ltd (cat no. c0618; Shanghai, China). Cells were maintained in RPMI-1640 medium (Gibco, Grand Island, New York, USA) supplemented with 10% fetal bovine serum (FBS; Gibco, Grand Island, New York, USA), 10,000 units/ml penicillin and 10,000 µg/ml streptomycin in a 5% CO_2_ incubator at 37°C according to the previous study [19].

### Cell transfection

hHSFs (1 × 10^6^ cells/well) were divided into 6-well plates and incubated for 24 h until 75% confluence. Short hairpin RNA (shRNA) against INHBA-AS1 (sh-INHBA-AS1-1 or sh-INHBA-AS1-2), scrambled negative control (shRNA-NC), miR-141-3p mimic (forward: 5ʹ-UAUUGCACAUUACUAAGUUGCA-3ʹ, reverse: 5ʹ-CAACUUAGUAAUGUGCAAUAUU-3ʹ), miR-141-3p inhibitor (5ʹ-UGCAACUUAGUAAUGUGCAAUA-3ʹ), mimic-NC (5ʹ-UUCUCCGAACGUGUCACUGUU-3ʹ) and inhibitor-NC (5ʹ-CAGUACUUUUGUGUAGUACAA-3ʹ), sh-MCL1-1, sh-MCL1-2 and sh-NC were provided by Genepharma (Shanghai, China). Transfection experiments were carried out by means of Lipofectamine® 3000 (Invitrogen, Carlsbad, CA, USA) according to the manufacturer’s instructions. Subsequent experiments were performed 24 h following transfection.

### Cell viability assay

Cell Counting Kit-8 (CCK-8; Beyotime, Shanghai, China) assay was performed for determining the viability of hHSFs according to the previous study [20]. 5 × 10^3^ cells were plated into the 96-well tissue culture plates and maintained at 37°C. At each of the desired time points, plates were cultured for 4  h after each well was added with 10 μL CCK-8 reagent. The optical density was determined at 450 nm with a microplate reader (Bio-Rad Laboratories, Richmond, CA, USA).

### Transwell migration assay

Transwell inserts (Corning, Corning, NY, USA) containing 8-μm permeable pores were used to evaluate migration capacity in this study. Briefly, a serum-free medium containing 2 × 10^5^ hHSFs covered the upper chamber. And the lower chamber was filled with RPMI-1640 medium supplemented in 10% FBS. Cells were allowed free migration across the porous filters for 48 h at 37°C. Afterward, 4% paraformaldehyde was applied for fixation of migrated cells. After cells being stained with crystal violet, they were counted by means of an inverted light microscope (Olympus Corporation, Japan) and the results were analyzed using ImageJ software (version 1.52r; National Institutes of Health; Bethesda, MD, USA).

### Wound healing assay

Cells of each group were collected and plated into 6-well plates. When the cells reached 80% confluence, a vertical line was gently drawn using a 10 μl pipette tip on cell monolayers. Subsequently, the floating debris were washed with phosphate buffer solution (PBS) and visualized under an inverted microscope (Olympus Corporation, Japan). The images were labeled as 0 h. The media was then replaced with serum-free medium, and cells were further cultured for 24 h. Cell migration was photographed with an inverted microscope (Olympus Corporation, Japan).

### Immunofluorescence staining

Treated cells were fixed in 4% paraformaldehyde for 20 min, followed by the addition of 0.05% Triton X-100 solution for 10 min. Cells were blocked with 5% normal goat serum and then incubated with a primary antibody (anti-Col I antibody, cat. no. 72026S). After incubation with DyLight™ 488-conjugated secondary antibody (Cell Signaling Technology, Boston, MA, USA), cells were stained with 4ʹ, 6-diamidino-2-phenylindole (Sigma-Aldrich; Merck KGaA). Images were captured under a fluorescence microscope (Olympus Corporation, Japan).

### Luciferase activity reporter assay

The LncBase (http://carolina.imis.athena-innovation.gr/diana_tools/web/index.php?r=lncbasev2%2Findex) and Starbase (http://starbase.sysu.edu.cn/) online bioinformatics software were used to predict the targets of INHBA-AS1 and miR-141-3p, and the results showed that miR-141-3p could be potentially targeted by INHBA-AS1 and that MCL1 was an identified a putative target of miR-141-3p. Dual luciferase activity reporter experiments were executed for verifying these combinations. Briefly, wild-type (WT) or mutant (MUT) INHBA-AS1 or MCL1 binding miR-141-3p was subcloned into a pGL3 basic vector (Promega Corporation, Madison, WI, USA). INHBA-AS1 WT/MUT or MCL1-3ʹ untranslated region (UTR) WT/MUT, miR-141-3p mimic or mimic-NC were then co-transfected with cells with Lipofectamine 3000 (Invitrogen, Carlsbad, CA, USA) following the manufacturer’s guide. Two days after transfection, the luciferase activities were analyzed with a Dual-Luciferase Reporter Assay Kit (Promega Corporation).

### RNA immunoprecipitation (RIP) assay

The interaction between INHBA-AS1 and miR-141-3p was further identified by Magna RIP Kit (Millipore, Bedford, MA), referring to the instructions of the manufacturer [21]. hHSFs were lysed with RNA lysis buffer (Beyotime, Shanghai, China). After the cells were lysed, the cell lysates were incubated with magnetic beads conjugated to anti-Argonaute2 (Ago2; cat. no. ab32381; Abcam Company, Cambridge, UK) or control IgG. After washing the unbound material on the magnetic beads with RIP buffer, the magnetic beads were resuspended with TRIzol Reagents to purify the bound RNAs. Then, performed reverse transcription-quantitative PCR (RT-qPCR) analysis to evaluate the level of extracted RNAs.

### RT-qPCR analysis

Total RNA was extracted using TRIzol reagent (Invitrogen, Carlsbad, CA, USA), and complementary DNA (cDNA) was synthesized with the PrimeScript RT Reagent Kit (Takara, Japan). Using cDNA as the template, the gene expression levels were analyzed by PCR conducted using iTaq™ Universal One-Step iTaq™ Universal SYBR® Green Supermix (Bio-Rad Laboratories, Inc.) on an ABI 7500 instrument (Applied Biosystems). The following primers pairs were used: INHBA-AS1 forward, 5ʹ-TATGCAGAGCCTTGGAAAGATGG-3ʹ and reverse, 5ʹ-TCCAGAAGCTCCTCATGGGATAAAG-3ʹ; MiR-141-3p forward, 5ʹ-TAACACTGTCTGGTAAAGATGG-3ʹ and reverse, 5ʹ-TGGTGTCGTGGAGTCG-3ʹ; Col III forward, 5ʹ-TTGAAGGAGGATGTTCCCATCT-3ʹ and reverse, 5ʹ-ACAGAC ACATATTTGGCATGGTT-3ʹ; Col IV forward, 5ʹ-CTGGTCCAAGAGGATTTCCA-3ʹ and reverse, 5ʹ-TCATTGCCTTGCACGTAGAG-3ʹ; α-SMA forward, 5ʹ-TCAAATACCCCATTGAACACGG-3ʹ and reverse, 5ʹ-GGTGCTCTTCAGGTGCTACA-3ʹ; MCL1 forward, 5ʹ-GGACATCAAAAACGAAGACG-3ʹ and reverse, 5ʹ-GCAGCTTTCTTGGTTTATGG-3ʹ; GAPDH forward, 5ʹ-ACAACTTTGGTATCGTGGAAGG-3ʹ and reverse, 5ʹ- GCCATCACGCCACAGTTTC-3ʹ; U6 forward, 5ʹ-GGAACGATACAGAGAAGATTAGC-3ʹ and reverse, 5ʹ-TGGAACGCTTCACGAATTTGCG-3ʹ. All primers were provided by Genepharma (Shanghai, China). The relative levels were quantitatively analyzed using the 2^−ΔΔCt^ method with GAPDH or U6 as an endogenous control [22].

### Western blotting

Total proteins in conditioned hHSFs cells were collected and lysed with radioimmunoprecipitation assay lysis buffer (Beyotime, Shanghai, China) 24 h after transfection. The concentration of protein was examined according to the bicinchoninic acid (BCA) kit instructions (Kaiji, China). Afterward, 40 μg of each sample were loaded and separated by a 10% sodium dodecyl sulfate-poly acrylamide gel electrophoresis (SDS-PAGE) gels. The protein was bind to the polyvinylidene fluoride (PVDF) membrane (Millipore) by electrophoretic. And then blocking the nonspecific sites on the membrane with 5% skimmed milk for 1.5 h at room temperature. After washing three times with 0.2% TBS-Tween 20, these blots were probed with specific primary antibodies and secondary antibody conjugated with horseradish peroxidase (cat. no. ab205718; Abcam Company, Cambridge, UK). Anti-Ki67 (cat. no. ab16667) antibody was purchased from Abcam Company (Cambridge, UK). Anti-Col III (cat. no. 30565S), anti-Col IV (cat. no. 50273S), anti-α-SMA (cat. no. 19,245 T), anti-MCL1 (cat. no. 94296S) and anti GAPDH (cat. no. 5174 T) antibodies were obtained from Cell Signaling Technology (Boston, MA, USA). Protein bands were visualized with enhanced chemiluminescence substrate (Pierce, USA) using chemiluminescence imaging equipment (Claremont, CA, USA). The relative intensity of target bands were semi-quantified using ImageJ software (version 1.52r; National Institutes of Health; Bethesda, MD, USA) and normalized by the intensity of GAPDH.

### Statistical analysis

Analysis of the data was carried out with GraphPad Prism 8.0 (GraphPad Software). Three biological replications were conducted for each experiment. Untreated cells were used as control. Results were presented as the mean ± standard deviation (SD). Student’s *t*-test was used to compare differences between two groups. Analysis of variance (ANOVA) with Tukey’s post hoc test was employed to analyze significance among multiple groups. *P*-values of <0.05 were taken as statistically significant.

## Results

### INHBA-AS1 is highly expressed in hHSFs and miR-141-3p is directly targeted by INHBA-AS1

**Figure 1.**
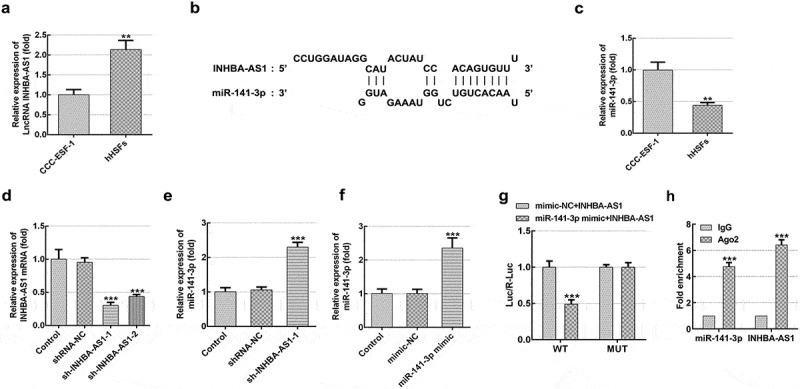
INHBA-AS1 is highly expressed in hHSFs and miR-141-3p is directly targeted by INHBA-AS1. (a) INHBA-AS1 expression in hHSFs and CCC-ESF-1 was examined using RT-qPCR. **P < 0.01 vs. CCC-ESF-1. (b) Binding region between INHBA-AS1 and miR-141-3p. (c) miR-141-3p level in hHSFs and CCC-ESF-1 was detected using RT-qPCR. **P < 0.01 vs. CCC-ESF-1. (d) INHBA-AS1 expression was measured using RT-qPCR after transfection with sh-INHBA-AS1-1 or sh-INHBA-AS1-2. (e) miR-141-3p level was examined using RT-qPCR when INHBA-AS1 was silenced. ***P < 0.001 vs. shRNA-NC. (f) RT-qPCR was used to evaluate miR-141-3p expression after transfection with miR-141-3p mimic. ***P < 0.001 vs. mimic-NC. (g) Relative luciferase activities were detected in hHSFs. ***P < 0.001 vs. mimic-NC+ INHBA-AS1. (h) RIP assay were conducted to measure the enrichment of INHBA-AS1 and miR-141-3p in the Ago2 immunoprecipitation and IgG-pellet. hHSFs, human hypertrophic scar fibroblasts; sh, short hairpin; NC, negative control; WT, wild-type; MUT, mutant

LncRNAs play significant roles in the progression of HS. INHBA-AS1 has been reported to be overexpressed in human hypertrophic scar tissues compared with the normal skin tissues by high-throughput RNA sequencing [18]. To study the roles of INHBA-AS1 in HS, the level of INHBA-AS1 in hHSFs and CCC-ESF-1 cells were firstly determined. As shown in Figure 1a, INHBA-AS1 level was markedly enhanced in hHSFs compared with the CCC-ESF-1 cells. Then, the online bioinformatics software found that miR-141-3p was potentially targeted by INHBA-AS1, and the binding site was displayed in Figure 1b. Results in Figure 1c show that miR-141-3p level was lower in hHSFs than CCC-ESF-1 cells. Additionally, INHBA-AS1 was silenced by transfection with sh-INHBA-AS1-1 or sh-INHBA-AS1-2. Cells transfected with sh-INHBA-AS1-1 displayed lower mRNA expression levels of INHBA-AS1 (Figure 1d). Therefore, sh-INHBA-AS1-1 was used to perform the following experiments. It is observable from Figure 1e that miR-141-3p expression was markedly elevated after INHBA-AS1 silencing compared to the shRNA-NC group. Subsequently, the luciferase activity reporter assay was utilized to confirm this association between INHBA-AS1 and miR-141-3p. Following miR-141-3p overexpression (figure 1f), the luciferase activity was remarkably reduced relative to that in cells co-transfected with mimic-NC and INHBA-AS1-WT (Figure 1g). Additionally, results of RIP assay revealed that miR-141-3p and INHBA-AS1 were greatly enriched compared with the IgG group (Figure 1h). These results implicate that INHBA-AS1 is notably increased in hHSFs and that miR-141-3p is directly targeted by INHBA-AS1.

### MiR-141-3p silencing markedly reverses the effects of INHBA-AS1-silencing on proliferation and migration of hHSFs

**Figure 2.**
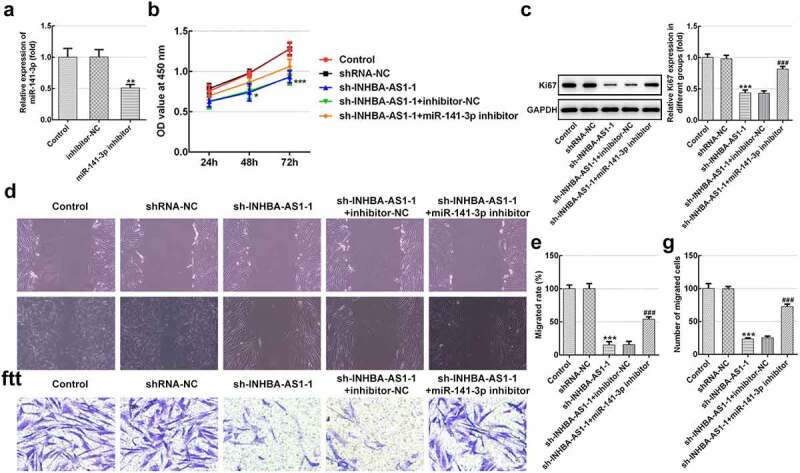
MiR-141-3p silencing markedly alleviates the inhibitory impact of INHBA-AS1-knockdown on proliferation and migration of hHSFs. (a) MiR-141-3p expression was tested using RT-qPCR after transfection with miR-141-3p inhibitor. **P < 0.01 vs. inhibitor-NC. (b) Cell viability was measured by a cell counting Kit-8 assay. (c) Western blot analysis was utilized for detecting the expression of Ki67. (d and e) Wound healing assay and (f and g) Transwell migration assay were performed to evaluate migration of hHSFs. ***P < 0.001 vs. mimic-NC. *P < 0.05, ***P < 0.001 vs. shRNA-NC; ^###^*P* < 0.001 vs. sh-INHBA-AS1-1+ inhibitor-NC. hHSFs, human hypertrophic scar fibroblasts; sh, short hairpin; NC, negative control

Afterward, rescue experiments were conducted to clarify the molecular mechanisms. As exhibited in Figure 2a, miR-141-3p level was noticeably decreased in hHSFs transfected with miR-141-3p inhibitor relative to the inhibitor-NC group. Afterward, cell viability was tested by means of a CCK-8 kit. Results in Figure 2b indicate that INHBA-AS1-knockdown dramatically inhibited cell viability by contrast with the shRNA-NC group, while miR-141-3p silencing ameliorated the impact of INHBA-AS1-downregulation on cell viability. Consistently, the expression of proliferation-related protein Ki67 displayed the same variation with cell viability (Figure 2c). Additionally, wound healing- (Figure 2d and e) and Transwell migration assays (figure 2f and g) revealed that INHBA-AS1-knockdown notably reduced the ability of cell migration as comparison to the transfection control group, which was enhanced following co-transfection with the miR-141-3p inhibitor. Through the above results we proved that INHBA-AS1 could modulate the proliferation and migration of hHSFs via targeting miR-141-3p.

### MiR-141-3p inhibitor restores the impact of INHBA-AS1-knockdown on the ECM deposition of hHSFs

**Figure 3.**
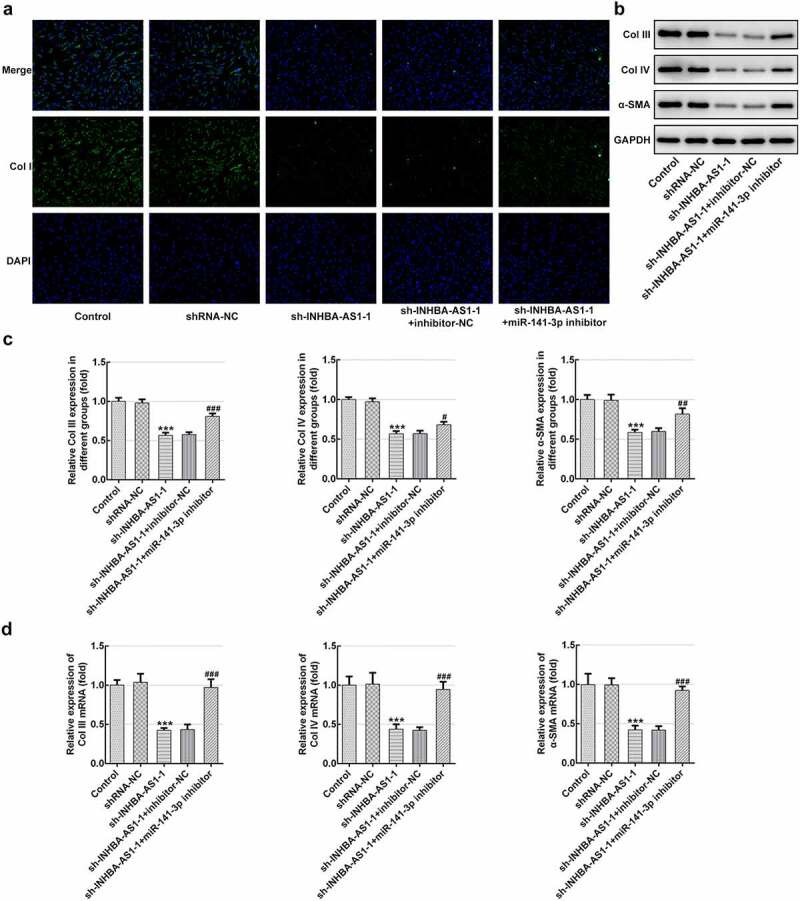
MiR-141-3p inhibitor restores the effects of INHBA-AS1-knockdown on the ECM deposition of hHSFs. (a) Expression of col I was measured by immunofluorescence assay. (b and c) The protein expression and (d) mRNA expression of Col III, Col IV and α-SMA were detected using western blot analysis and RT-qPCR, respectively. ***P < 0.001 vs. shRNA-NC; ^#^*P* < 0.05, ^##^*P* < 0.01, ^###^*P* < 0.001 vs. sh-INHBA-AS1-1+ inhibitor-NC. hHSFs, human hypertrophic scar fibroblasts; sh, short hairpin; NC, negative control; Col I, collagen I; Col III, collagen III; Col IV, collagen IV; α-SMA, alpha smooth muscle actin

To analyze the role of INHBA-AS1 and miR-141-3p on ECM accumulation in the formation of HS, Col I expression was examined using immunofluorescence staining. As exhibited in Figure 3a, INHBA-AS1 silencing remarkably decreased the level of Col I relative to the shRNA-NC group. Co-transfection with sh-INHBA-AS1-1 and miR-141-3p inhibitor restored Col I expression by contrast with the sh-INHBA-AS1-1+ inhibitor-NC group. Concurrently, the protein expression (Figure 3b and c) and mRNA expression (Figure 3d) of Col III, Col IV and α-SMA was markedly reduced after INHBA-AS1-downregulation, which was mitigated by miR-141-3p silencing. To conclude, the data suggest that INHBA-AS1 regulates ECM deposition of hHSFs by targeting miR-141-3p.

### MCL1 is a direct target gene of miR-141-3p

**Figure 4.**
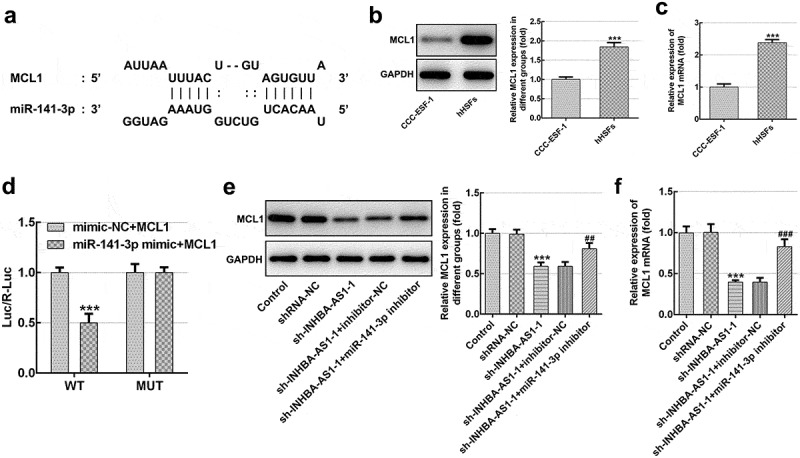
MCL1 is a direct target gene of miR-141-3p. (a) Binding region between miR-141-3p and MCL1. (b) MCL1 protein expression and (c) mRNA expression was tested using western blot analysis and RT-qPCR, respectively. ***P < 0.001 vs. CCC-ESF-1. (d) Relative luciferase activities were detected in hHSFs. ***P < 0.001 vs. mimic-NC+MCL1. (e) MCL1 protein level and (f) mRNA level were determined using western blot analysis and RT-qPCR, respectively. ***P < 0.001 vs. shRNA-NC; ^##^*P* < 0.01, ^###^*P* < 0.001 vs. sh-INHBA-AS1-1+ inhibitor-NC. hHSFs, human hypertrophic scar fibroblasts; sh, short hairpin; NC, negative control; WT, wild-type; MUT, mutant

To further explore the possible regulatory mechanisms of INHBA-AS1 and miR-141-3p on HS, the Starbase online bioinformatics software was used to the prediction of the targets of miR-141-3p. It was noticed that MCL1 could be a potential target gene of miR-141-3p (Figure 4a). Subsequently, significantly upregulated MCL1 expression was observed in hHSFs in comparison to CCC-ESF-1 cells (Figure 4b and c). Results of luciferase activity reporter assay presented in Figure 4d indicates that the luciferase activity was notably decreased compared with that in cells co-transfected with mimic-NC and MCL1-WT. In addition, MCL1 level was decreased remarkably in the sh-INHBA-AS1 group, while co-transfection with miR-141-3p inhibitor restored the level of MCL1 (Figure 4e and f). Collectively, above results suggest that MCL1 serves as a direct target gene of miR-141-3p.

### MCL1-knockdown alleviates the proliferation, migration and ECM deposition in hHSFs

**Figure 5.**
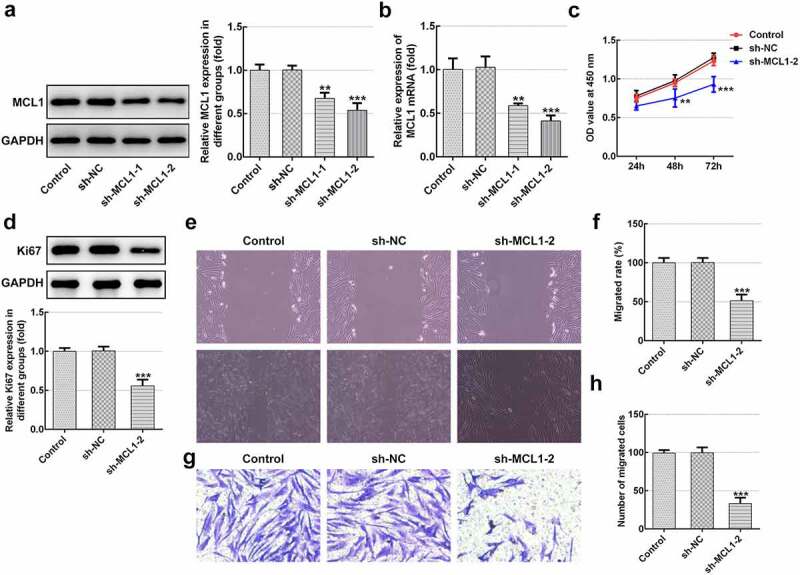
MCL1-knockdown alleviates the proliferation and migration of hHSFs. MCL1 expression was examined using (a) western blot analysis and (b) RT-qPCR, respectively. (c) Cell viability was tested using a cell counting kit-8 assay. (d) Ki67 expression was evaluated using western blot analysis. (e) Representative images and (f) relative quantification of cell migration measured by wound healing assay at different time points. (g) Migration activity of hHSFs was assessed using transwell migration assay. (h) Number of migrated cells was quantified. **P < 0.01, ***P < 0.001 vs. sh-NC. hHSFs, human hypertrophic scar fibroblasts; sh, short hairpin; NC, negative control

**Figure 6.**
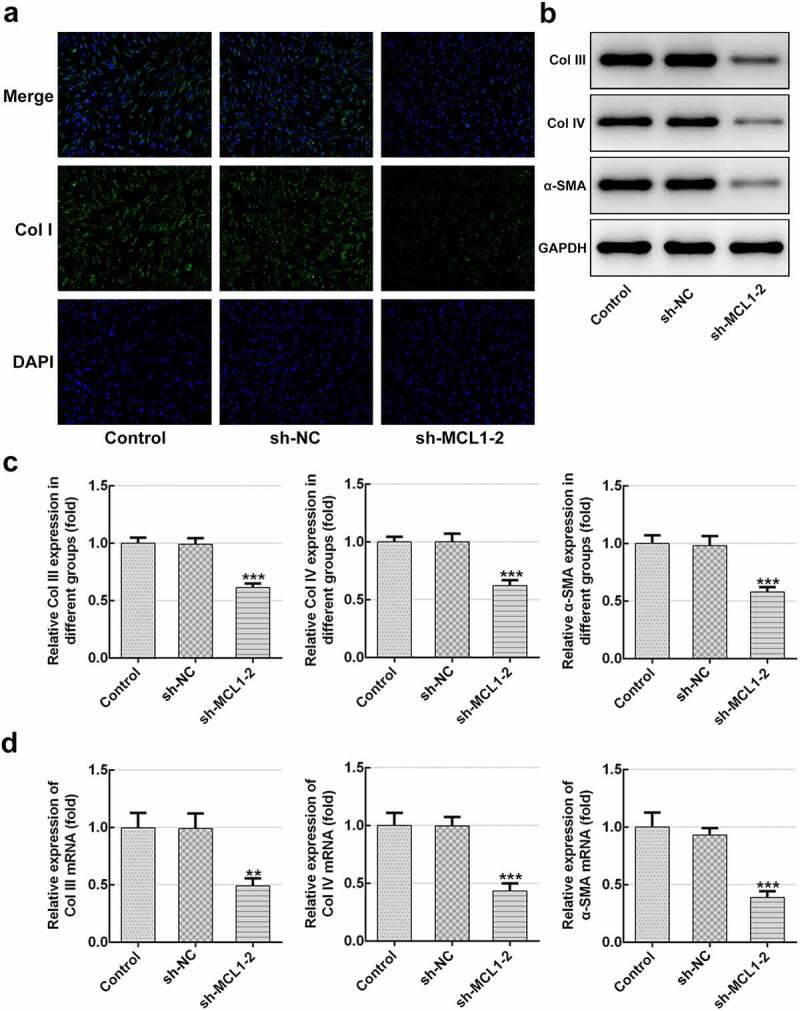
MCL1-knockdown mitigates the ECM deposition in hHSFs. (a) Expression of col I was detected using immunofluorescence assay. (b and c) The protein expression and (d) mRNA expression of Col III, Col IV and α-SMA were detected using western blot analysis and RT-qPCR, respectively. **P < 0.01, ***P < 0.001 vs. sh-NC.hHSFs, human hypertrophic scar fibroblasts; sh, short hairpin; NC, negative control; Col I, collagen I; Col III, collagen III; Col IV, collagen IV; α-SMA, alpha smooth muscle actin

Then, the effects of MCL1 on the cellular functions of hHSFs were explored. As displayed in Figure 5a and b, MCL1 expression was notably downregulated after transfection with sh-MCL-1 or 2. And cells transfected with sh-MCL-2 were chosen to conduct the following experiments. Dramatically decreased cell viability (Figure 5c), Ki67 level (Figure 5d) and cell migration (Figure 5e–h) were noticed in the MCL1 silencing group as comparison to the negative control group. Additionally, it can be observed from Figure 6a–d that the expression of Col I, Col III, Col IV and α-SMA was remarkably decreased after MCL1-knockdown by contrast to the sh-NC group. To conclude, the experimental results and the findings mentioned above provide evidence that MCL1 silencing attenuates the proliferation, migration and ECM deposition in hHSFs.

## Discussion

As the key effector cells in the process of wound healing, fibroblasts exhibit excessive hypercellularity, increased migration and superabundant ECM accumulation during the formation of HS [23]. Dysfunction of lncRNAs may aggravate the progression of skin fibrosis [24,25]. Findings of this study demonstrated high level of INHBA-AS1 in hHSFs, and that INHBA-AS1 silencing exhibited efficiently the proliferation, migration and ECM synthesis in hHSFs via regulating miR-141-3p/MCL1 axis.

It is generally well known that lncRNAs act as ceRNAs to regulate the progression of disease by sponging specific miRNAs, indirectly modulating mRNA expression [26,27]. It has been reported that INHBA-AS1 accelerates cell growth, invasion and migration of oral squamous cell carcinoma via targeting miR-143-3p [17]. Emerging evidence supports that INHBA-AS1 is notably enhanced in human hypertrophic scars tissues, but the detailed functions and mechanisms remain to be elucidated [18]. The LncBase online bioinformatics software predicted that miR-141-3p was putatively targeted by INHBA-AS1, which was confirmed by a luciferase activity reporter- and RIP assays in the present study. Report has demonstrated previously that miR-141-3p silencing attenuated the positive effects of TUG1 deletion on the proliferation and migration of oxidized low-density lipoprotein-stimulated vascular smooth muscle cells [28]. LncRNA SNHG16 promotes proliferation and fibrogenesis by downregulating miR-141-3p expression and upregulating CCND1 expression in diabetic nephropathy [29]. Importantly, miR-141-3p suppresses fibroblast proliferation and migration via targeting GAB1 in keloids [30]. Mounting evidence suggests that excessive collagen synthesis is a typical pathological feature of HS formation [31]. Collagens, mainly including Col I, Col III and Col IV, are the most important extracellular matrix structural protein [32]. Additionally, α-SMA was a crucial regulator of ECM metabolism in several tissues [33]. Previous study reported that the levels of the above-mentioned factors in hHSF cells and HS tissues are remarkably elevated [34]. This study revealed that INHBA-AS1-knockdown inhibits the proliferation, migration and ECM deposition of hHSFs, and these positive effects could be partly restored by miR-141-3p silencing. These observations demonstrated that INHBA-AS1 regulates the formation of HS by targeting miR-141-3p.

To explore the biological mechanisms of the role of miR-141-3p in HS formation, prediction program was conducted to identify the putative targets of miR-141-3p. The luciferase reporter assay demonstrated that MCL1 is a direct target of miR-141-3p. Mukherjee et al. demonstrated that MCL1 inhibitors plus Navitoclax can synergistically kill difficult-to-treat melanoma cells [35]. Research has proposed that MCL1 is involved in inducing synergistic inhibitory effects on Fas-mediated apoptosis in model vitiligo model [36]. It has been well reported that the level of MCL1 was increased in pathological scars, and that overexpression of MCL1 exerts a crucial effect on the proliferation of fibroblasts and the pathogenesis of pathological scars [37]. This study suggested that MCL1 level was dramatically upregulated in hHSFs, and INHBA-AS1-knockdown significantly reduced the level of MCL1 while co-transfection with miR-141-3p inhibitor restored the level of MCL1. Additionally, MCL1 deletion mitigated proliferation, migration and ECM synthesis. These data provide evidence that miR-141-3p mediates the formation of HS by targeting MCL1.

## Conclusion

Taken together, the present study demonstrated that INHBA-AS1 regulates proliferation, migration and ECM deposition at least partially by targeting miR-141-3p/MCL1 axis, which is the first to clarify the underlying regulatory mechanisms of INHBA-AS1 in the development of HS. Our findings present novel insights into the mechanism of HS physiology and new strategies for developing therapeutic interventions. However, whether similar effects may be observed in clinical situations involving HS formation remains to be determined. Therefore, further experiments will be performed in the future investigations.

## Data Availability

The datasets generated and/or analyzed during the current study are available on reasonable request.

## Highlights

(1) INHBA-AS1 is highly expressed in human hypertrophic scar fibroblasts (hHSFs).

(2) INHBA-AS1 acts as a sponge of miR-141-3p in hHSFs.

(3) MCL1 is a direct target gene of miR-141-3p in hHSFs, and positively regulated by INHBA-AS1.

(4) Silencing of INHBA-AS1 suppresses the progression of hHSFs via regulating miR-141-3p/MCL1 axis.
